# Complete quenching phenomenon for a parabolic *p*-Laplacian equation with a weighted absorption

**DOI:** 10.1186/s13660-018-1841-5

**Published:** 2018-09-20

**Authors:** Liping Zhu

**Affiliations:** College of Science, Xi’an University of Architecture & Technology, Xi’an, China

**Keywords:** 35D30, 35K55, 35K92, Parabolic *p*-Laplacian, Complete quenching, Weighted absorption

## Abstract

Throughout this paper, we mainly consider the parabolic *p*-Laplacian equation with a weighted absorption $u_{t}-\operatorname{div} (|\nabla u|^{p-2}\nabla u )=-\lambda |x|^{\alpha} {\chi}_{\{u>0\}}u^{-\beta}$ in a bounded domain $\Omega\subseteq\mathbb{R}^{n}$ ($n\geq1$) with Lipschitz continuous boundary subject to homogeneous Dirichlet boundary condition. Here $\lambda>0$ and $\alpha>-n$ are parameters, and $\beta\in(0,1)$ is a given constant. Under the assumptions $u_{0}\in W_{0}^{1,p}(\Omega)\cap L^{\infty}(\Omega)$, $u_{0}\geq0$ a.e. in Ω, we can establish conditions of local and global in time existence of nonnegative solutions, and show that every global solution completely quenches in finite time a.e. in Ω. Moreover, we give some numerical experiments to illustrate the theoretical results.

## Introduction

In this paper, we mainly study the following initial-boundary value problem for the *p*-Laplacian equation

1.1 $$ \textstyle\begin{cases} u_{t}-\Delta_{p}u =-\lambda|x|^{\alpha} {\chi}_{\{u>0\} }u^{-\beta}, & x\in\Omega, t>0, \\ u =0, & x\in\partial\Omega, t>0, \\ u(x,0) =u_{0},& x\in\Omega, \end{cases} $$

where $\Omega\subseteq\mathbb{R}^{n}$ ($n\geq1$) is a bounded domain with Lipschitz continuous boundary *∂*Ω, $\Delta_{p} u=\operatorname{div} (|\nabla u|^{p-2}\nabla u )$, $1< p<\infty$, and $0<\beta<1$, $\lambda>0$, $\alpha>-n$; $\chi_{\{u>0\}}$ is the characteristic function on $\{u>0\}$, i.e.,

1.2 $$ \chi_{\{u>0\}}= \textstyle\begin{cases} 1,& u>0, \\ 0,& u\leq0. \end{cases} $$

In the present paper, we suppose that $u_{0}$ satisfies the following assumptions:

1.3 $$ u_{0}\geq0 \quad \mbox{a.e. in } \Omega \quad \mbox{and} \quad u_{0}\in W_{0}^{1,p}(\Omega)\cap L^{\infty}(\Omega). $$

For convenience, let ${\chi}_{\{u>0\}}u^{-\beta}=0$ whenever $u=0$, and define $Q_{T}=\Omega\times(0,T)$, $\Gamma_{T}=\partial\Omega \times(0,T)$.

When $p=2$ in (1.1), the semilinear parabolic equations with singular absorptions have been extensively studied, we refer to [23–25, 31] and the references therein. Guo et al. [26–28, 38] studied the weighted singular parabolic problem

1.4 $$ \textstyle\begin{cases} u_{t}-\Delta u=\frac{\lambda f(x)}{(1-u)^{2}}, &Q_{T}, \\ u(x,t)=0, & \Gamma_{T}, \\ u(x,0)=u_{0}\geq0, & \Omega, \end{cases} $$

where $\Omega\subseteq\mathbb{R}^{n}$ ($n\geq1$) and $\lambda>0$ is a parameter. When $n=1\mbox{ or }2$, (1.4) models a simple electrostatic Micro-Electro-Mechanical-System (MEMS) device consisting of a thin dielectric elastic membrane. In this model, the dynamic solution *u* characterizes the dynamic deflection of the elastic membrane. When a voltage *λ* is applied to the surface of the membrane, the membrane deflects towards the ceiling plate and a snap-through may occur when it exceeds a certain critical value $\lambda^{*}$ (pull-in voltage). This creates a so-called “pull-in instability,” which greatly affects the design of many devices. In order to achieve better MEMS designs, the material properties of the membrane can be technologically fabricated with a spatially varying dielectric permittivity profile $f(x)$. We refer to [17, 38] and the references therein for more detailed discussions on MEMS devices. Guo et al. [18, 21] studied the stationary problem (1.4), and gave the existence and some properties of the pull-in voltage $\lambda=\lambda^{\ast}$.

Moreover, Guo [26] studied the problem (1.4) for $f(x)=|x|^{\alpha}$, $\alpha>0$, and Ω being the unit ball in $\mathbb{R}^{n}$ ($n\geq2$). Under certain conditions of $\lambda, n$ and *α*, Guo showed the stability of the minimal compact stationary solution and the instability of the singular stationary solution of (1.4), respectively. Guo and Wei [29] studied the Cauchy problem with a singular nonlinearity $u_{t}=\Delta u-u^{-\nu }$ with $\nu>0$ and proved that the problem has a global classical solution, and studied the properties of positive radial solutions of the steady state. More generally, Castorina et al. [5] studied the *p*-MEMS equation $-\Delta_{p}u={\lambda}/{(1-u)^{2}}$ in a ball and proved the uniqueness of semi-stable solutions and stability of minimal solutions for $1< p\leq2$.

For the *p*-Laplacian equation with absorption

1.5 $$ u_{t}=\Delta_{p}u-\beta u^{q},\quad \beta, q>0, $$

we known that near $u=0$ the absorption is strong when $q<1$, and the absorption is weak when $q\geq1$. This problem appears in the theory of quasiregular and quasiconformal mappings, stochastic control and non-Newtonian fluids, etc. In the non-Newtonian theory, the quantity *p* is a characteristic of the medium. Media with $p>2$ are called dilatant fluids while those with $p<2$ are called pseudoplastics. If $p=2$, they are called Newtonian fluids. For example, we refer to [6–8, 20].

Galaktionov and Vazquez [20] systematically studied the properties of several equations, such as complete or incomplete blowup and extinction. Firstly, they studied the problem $u_{t}=\Delta u^{m}+u^{q}$, with $m>1$, $q>1$. Assuming that $p>1$, $m>(n-2)/n$, and $n\geq2$, they proved that when $p+m\leq2$ incomplete blowup always occurs; when $p+m>2$, the radially symmetric solutions always blow up completely. Secondly, they studied the equation

$$u_{t}=\Delta_{p}u+u^{q}, \quad p>1, q>1, $$

and showed that blowup is always incomplete if $q\leq1/(p-1)$, and complete if $1/(p-1)< q\leq q_{s}(p,n)=[n(p-1)+p]/(n-p)^{+}$. Lastly, assumed that the initial function $u_{0}=u_{0}(r)$ is strictly positive, bounded away from zero and has an inverse bell-shaped form. Then they studied another kind of singularity of the equation $u_{t}=\Delta u^{m}-u^{-q}$, with $m>1$, $q>0$, and proved that extinction is complete if and only if $q+m\leq0$. They also studied equation with the *p*-Laplacian operator

$$u_{t}=\Delta_{p}u-u^{-q}, \quad p>1, q>0. $$

Under the given assumptions on $u_{0}(r)$, they showed that extinction is complete if and only if $q\geq1$.

There are some recent works on local and global existence, gradient estimates, blowup and extinction of the *p*-Laplacian equations. We refer to [32, 35, 44, 45] for the nonlinear absorption and source, nonlinear gradient absorption or source, and [9, 10, 22] for singular absorptions. Also, we refer to [46, 47] for the semilinear equations with an exponential source. When $\alpha=0$, equation (1.1) is known as a limit model of a class of problems arising in Chemical Engineering corresponding to enzymatic kinetics and hetergeneous catalyst of Langmuir–Hinshelwood type, see [3, 9, 12, 15, 22, 39, 43] and references therein. Under the Dirichlet boundary condition, problem (1.1) of $p=2$ has been studied by many authors, we refer to [14, 19, 30] and the references therein. The Cauchy problem for equation (1.1) was studied by Phillips [39]. Winkler [42] studied the nondivergent parabolic equations with singular absorption. Under certain conditions, Giacomoni et al. [22] showed that problem (1.1) has a global in time bounded weak solution. Moreover, every weak solution *u* completely quenches in a finite time $T_{\ast}$, i.e., $u(\cdot,t)=0$ a.e. in Ω for all *t* beyond $T_{\ast}$.

Due to the singular absorption, the solution *u* of (1.1) may quench in finite time on one set with nonzero measure, even if the initial datum is strictly positive (see [11–13, 37]). Davila and Montenegro [11–13] have studied the semilinear problem (1.1) with $p=2$ and $\alpha=0$ under the assumptions $u_{0}\geq0$ a.e. in Ω and $u_{0}\in L^{\infty}(\Omega)\cap C(\Omega)$. Moreover, under certain stronger conditions on $u_{0}$, Montenegro [37] showed that the solution *u* of (1.1) with $p=2$ and $\alpha =0$ may quench completely.

Motivated by the above analytic results and observations, our interest is to study the weighted problem (1.1) with $1< p<\infty$ and $\alpha\neq0$. We first show that the weak solution exists in an arbitrary time interval under the conditions $\alpha>\max \{ -{n(p+\beta-1)}/{p}, -{n}/{2} \}$, $\lambda<{\lambda_{1} p}/(1-\beta)$, where $\lambda_{1}$ is the first eigenvalue of the Dirichlet problem for the *p*-Laplace operator (see [36]):

1.6 $$ \lambda_{1}:=\inf \biggl\{ \int_{\Omega} \vert \nabla v \vert ^{p}\,dx: v\in W_{0}^{1,p}(\Omega), \int_{\Omega}|v|^{p}\,dx=1 \biggr\} . $$

Next, we show that the global solution completely quenches in the finite time $T_{\ast}$, and then estimate $T_{\ast}$ through $\|u_{0}\| _{\infty,\Omega}$, $\|u_{0}\|_{2,\Omega}$, *n*, *p*, *α*, *λ* and $\lambda_{1}$.

To prove the main results, we organized the paper as follows: We give the definition of weak solutions and main results in Sect. 2. In Sect. 3, using Faedo–Galerkin method, we prove that weak solutions exist globally in time. Finally, we prove that the solution is uniformly bounded under conditions (1.3). In Sect. 4, we show that the global solution completely quenches in finite time, which is based on the analysis of an ordinary differential inequality satisfied by the function $\|u(x,t)\|_{2,\Omega}$. In this section, we make use of Gagliardo–Nirenberg interpolation inequality with weights (see Lemma 4.1 below or [33])

$$\bigl\Vert |x|^{\gamma}D^{j}u \bigr\Vert _{L^{r}} \leq c \bigl\Vert |x|^{\alpha}D^{m}u \bigr\Vert ^{a}_{L^{p}} \bigl\Vert |x|^{\beta}u \bigr\Vert ^{1-a}_{L^{q}}, $$

where the constants *γ*, *j*, *r*, *α*, *m*, *a*, *p*, *β* and *q* are restricted to certain ranges. In Sect. 5, we verify the correctness of theoretic results through numerical examples.

## Definition of weak solutions and main results

Define

$$\mathscr {U}:= \bigl\{ v\in L^{\infty}\bigl(0,T;W_{0}^{1,p}( \Omega)\bigr)\cap L^{\infty}(\Omega) | v_{t}\in L^{2}(Q_{T}) \bigr\} . $$

For convenience, we denote $u(t):=u(x,t)$ a.e. in Ω, and use $z=(x,t)$ for the points of $Q_{T}$. First, we give the definition of weak solutions of problem (1.1).

### Definition 2.1

The function $u(x,t)$ is called a weak solution of (1.1) if it satisfies

1. $u\in \mathscr {U}\cap C ([0,T];L^{2}(\Omega) )$, $u\geq0$ a.e. in $Q_{T}$;
2. $|x|^{\alpha}\chi_{\{u>0\}}u^{-\beta}\varphi\in L^{1}(Q_{T})$ holds for every test function $\varphi\in \mathscr {U}$, and
  $$\int_{Q_{T}}\partial_{t}u\cdot\varphi \,dz+ \int_{Q_{T}}|\nabla u|^{p-2}\nabla u\cdot\nabla\varphi \,dz +\lambda \int_{Q_{T}}|x|^{\alpha}\chi_{\{u>0\}}u^{-\beta} \varphi \,dz=0; $$
3. $u(x,0)=u_{0}$ a.e. in Ω.

Next, we give the main results of this paper.

### Theorem 2.1

*If* $u_{0}$ *satisfies conditions* (1.3), *then there exists a* $T^{\ast}>0$ *such that for every* $T< T^{\ast}$ *equation* (1.1) *has at least one weak solution*, *which satisfies the following energy relations*:

2.1 $$ \frac{1}{2} \bigl\Vert u(t_{2}) \bigr\Vert ^{2}_{2,\Omega}-\frac{1}{2} \bigl\Vert u(t_{1}) \bigr\Vert ^{2}_{2,\Omega}+ \int_{t_{1}}^{t_{2}} \int_{\Omega} \vert \nabla u \vert ^{p}\,dz +\lambda \int_{t_{1}}^{t_{2}} \int_{\Omega}|x|^{\alpha}u^{1-\beta}\,dz=0 $$

*for every* $t_{1}$, $t_{2}\in[0,T]$, *and*

2.2 $$\begin{aligned}& \Vert \partial_{t}u \Vert ^{2}_{2,\Omega}+ \frac{1}{p} \bigl\Vert \nabla u(t) \bigr\Vert ^{p}_{p,\Omega}+ \frac{\lambda}{1-\beta} \int_{\Omega }|x|^{\alpha}u^{1-\beta}(t)\,dx \\& \quad \leq\frac{1}{p} \Vert \nabla u_{0} \Vert ^{p}_{p,\Omega}+\frac{\lambda }{1-\beta} \int_{\Omega}|x|^{\alpha}u_{0}^{1-\beta} \,dx \end{aligned}$$

*for almost every* $t\in(0,T)$.

### Theorem 2.2

*Let the assumptions of Theorem *2.1 *be satisfied*. *Problem* (1.1) *has a bounded global weak solution* $u\in \mathscr {U}$ *provided that*

$$\alpha>\max \biggl\{ -\frac{n(p+\beta-1)}{p}, -\frac{n}{2} \biggr\} ,\qquad \lambda< \frac{\lambda_{1}p}{1-\beta}. $$

*Moreover*, *every weak solution* *u* *completely quenches in finite time*, *i*.*e*., *there exists a* $T_{\ast}>0$, *depending on* *p*, *n*, $|\Omega|$, *λ*, $\lambda_{1}$ (*defined as* (1.6)), $\|u_{0}\|_{2,\Omega }$, $\|u\|_{\infty,\Omega}$, *such that*

$$\forall t>T_{\ast}, \quad u(t)=0 \quad \textit{a.e. in } \Omega. $$

## Global weak solutions

For problem (1.1) with $\alpha=0$, the existence of local in time weak solutions can be obtained by studying the regularization equation and proving the uniform gradient estimates, and then passing the parameter to a limit. We refer to [9, 10, 22] for the details of proof, and Theorem 2.1 can be derived in a similar manner to [22, Theorem 2.1] (see also [10, Theorem 2] for the degenerate case of $p>2$ and $n=1$).

Here we are mainly interested in the asymptotic behavior of nonnegative and global solutions of the weighted problem (1.1). However, the equation is singular at $x=0$ for $-n<\alpha<0$. In fact, the solutions can be approximated, if necessary, by those satisfying the regularized equation $u_{t}-\Delta_{p} u=-\lambda(|x|+\epsilon)^{\alpha }{\chi}_{\{u>0\}}u^{-\beta}$ with the same initial-boundary value conditions and taking the limit $\epsilon\rightarrow0^{+}$.

To prove Theorem 2.2, under weaker assumptions on the data, we first consider the weaker regularity on the solutions and define the function space

$$\mathscr {W}:= \bigl\{ v\in L^{p} \bigl(0,T;W_{0}^{1,p}( \Omega) \bigr) |v_{t}\in L^{p'} \bigl(0,T;W^{-1,p'}( \Omega) \bigr), {1}/{p}+{1}/{p'}=1 \bigr\} . $$

### Theorem 3.1

*Assume* $u_{0}\in L^{2}(\Omega)$, *then* (1.1) *has a global in time weak solution if* $\alpha>\max \{-\frac{n(p+\beta-1)}{p},-\frac {n}{2} \}$, $\lambda<\frac{\lambda_{1}p}{1-\beta}$.

### Proof

We use the classical Faedo–Galerkin method for the parabolic equations (see [2, 34]) to prove this theorem. Here we just give a brief proof.

Assume that $\{\psi_{k} \}$ is an orthonormal basis of $L^{2}(\Omega)$, which is composed of the eigenfunctions of the operator

$$(\psi_{k},w )_{H_{0}^{s}(\Omega)}=\lambda_{k}(\psi _{k},w)_{2,\Omega}, \quad \forall w\in H_{0}^{s}( \Omega), s\geq1+n \biggl(\frac{1}{2}-\frac{1}{p} \biggr). $$

Then the solutions of (1.1) can be written as

3.1 $$ u^{(m)}(z)=\sum_{k=1}^{m}c_{k}^{(m)}(t) \psi_{k}(x), $$

where $c_{k}^{(m)}(t)$ are defined by the following equality:

3.2 $$ \bigl(\partial_{t}u^{(m)},\psi_{k} \bigr)_{2,\Omega}=- \bigl( \bigl\vert \nabla u^{(m)} \bigr\vert ^{p-2}\nabla u^{(m)},\nabla\psi_{k} \bigr)_{2,\Omega}- \bigl(\lambda|x|^{\alpha}\bigl(u^{(m)} \bigr)^{-\beta },\psi_{k} \bigr)_{2,\Omega}, $$

$k=1, \ldots, m$. From the above relations we obtain

$$\frac{1}{2} \bigl\Vert u^{(m)} \bigr\Vert ^{2}_{2,\Omega} \bigg|_{t=0}^{t=\tau }+ \int_{Q_{T}} \bigl[ \bigl\vert \nabla u^{(m)} \bigr\vert ^{p}+\lambda|x|^{\alpha}\bigl(u^{(m)} \bigr)^{1-\beta} \bigr]\,dz=0. $$

So we can derive the following inequality, by using Young’s inequality:

3.3 $$\begin{aligned}& \frac{1}{2} \bigl\Vert u^{(m)} \bigr\Vert ^{2}_{2,\Omega} \bigg|_{t=0}^{t=\tau }+ \int_{Q_{T}} \bigl\vert \nabla u^{(m)} \bigr\vert ^{p}\,dz \\& \quad \leq \biggl\vert \frac{1}{2} \bigl\Vert u^{(m)} \bigr\Vert ^{2}_{2,\Omega} \bigg|_{t=0}^{t=\tau}+ \int_{Q_{T}} \bigl\vert \nabla u^{(m)} \bigr\vert ^{p}\,dz \biggr\vert = \int_{Q_{T}}\lambda|x|^{\alpha}\bigl(u^{(m)} \bigr)^{1-\beta}\,dz \\& \quad \leq\frac{\lambda}{2} \int_{Q_{T}}|x|^{2\alpha}\,dz+\frac{\lambda }{2} \int_{Q_{T}} \bigl(u^{(m)} \bigr)^{2(1-\beta)}\,dz \\& \quad \leq\frac{\lambda}{2} \int_{Q_{T}}|x|^{2\alpha}\,dz+\frac{\lambda }{2}(1-\beta) \int_{Q_{T}} \bigl(u^{(m)} \bigr)^{2}\,dz+ \frac{\lambda \beta}{2}|\Omega|. \end{aligned}$$

We can now use Gronwall’s inequality to estimate the function $\| u^{(m)}(\cdot,t) \|^{2}_{2,\Omega}$, if *α* satisfies the condition $\alpha>-\frac{n}{2}$.

On the other hand, we can obtain the following inequality, by using Hölder’s and Young’s inequalities and the definition of $\lambda_{1}$:

3.4 $$\begin{aligned}& \int_{Q_{T}}\lambda|x|^{\alpha}\bigl(u^{(m)} \bigr)^{1-\beta}\,dz \\& \quad \leq \int_{Q_{T}}\frac{\lambda(p+\beta-1)}{p}|x|^{\frac{\alpha p}{p+\beta-1}}\,dz+ \int_{Q_{T}}\frac{\lambda(1-\beta)}{p} \bigl(u^{(m)} \bigr)^{p}\,dz \\& \quad \leq \frac{\lambda(p+\beta-1)}{p} \int_{Q_{T}}|x|^{\frac{\alpha p}{p+\beta-1}}\,dz+\frac{\lambda(1-\beta)}{\lambda_{1}p} \int _{Q_{T}} \bigl(\nabla u^{(m)} \bigr)^{p} \,dz. \end{aligned}$$

Using Gronwall’s inequality again, we obtain a priori estimates of $\|\nabla u^{(m)}(\cdot,t) \|^{p}_{p,\Omega}$, if *α*, *λ* satisfy the conditions $\frac{\alpha p}{p+\beta-1}>-n$, $\frac{\lambda(1-\beta)}{\lambda_{1}p}<1$. So *α* and *λ* need to satisfy the conditions of $\alpha>\max \{-\frac {n(p+\beta-1)}{p},-\frac{n}{2} \}$, $\lambda<\frac{\lambda _{1}p}{1-\beta}$.

Since the sequence of functions $\{u^{(m)} \}$ is uniformly bounded about a priori estimates, applying the compactness results of [40], we can extract a subsequence which converges to a weak solution *u* of the problem (1.1):

$$\begin{aligned}& u^{(m)}\rightharpoonup u \quad \mbox{in } L^{p} \bigl(0,T;W_{0}^{1,p}(\Omega) \bigr),\qquad u^{(m)} \rightarrow u \quad \mbox{a.e. in } Q_{T}, \\& \partial_{t}u^{(m)}\rightharpoonup\partial_{t} u \quad \mbox{in } L^{p'} \bigl(0,T;W^{-1,p'}(\Omega) \bigr), \\& \bigl\vert \nabla u^{(m)} \bigr\vert ^{p-2}\nabla u^{(m)}\rightharpoonup \vert \nabla u \vert ^{p-2}\nabla u \quad \mbox{in } L^{p'}(Q_{T}), \end{aligned}$$

as $m\rightarrow\infty$. Here we refer to Barbu [4, Lemma 4.1 and Theorem 4.2] (or [34]) for the continuous embedding $\mathscr {W}\hookrightarrow C ([0,T];L^{2}(\Omega) )$. Also, for $v_{1}, v_{2}\in \mathscr {W}$, $t_{1}, t_{2}\in[0,T]$, we get

$$\int_{\Omega}v_{1}(t_{2})v_{2}(t_{2}) \,dx- \int_{\Omega }v_{1}(t_{1})v_{2}(t_{1}) \,dx= \int_{t_{1}}^{t_{2}} \int_{\Omega} (v_{2}\partial _{t}v_{1}+v_{1} \partial_{t}v_{2} )\,dz. $$

In particular, when $v_{1}=v_{2}$, we have

$$\frac{1}{2} \bigl\Vert v_{1}(t_{2}) \bigr\Vert ^{2}_{2,\Omega}-\frac{1}{2} \bigl\Vert v_{1}(t_{1}) \bigr\Vert ^{2}_{2,\Omega}= \int_{t_{1}}^{t_{2}} \int_{\Omega }v_{1}\partial_{t}v_{1} \,dz. $$

 □

### Theorem 3.2

*Assume that* $u_{0}\in L^{\infty}$, $u_{0}\geq0$ *a*.*e*. *in* Ω, *then there exist* $M>0$ *and* $T^{\ast}>0$ *such that a solution* *v* *of* (1.1) *satisfies* $0\leq v\leq M$ *a*.*e*. *in* $Q_{T}$ *for* $T< T^{\ast}$.

### Proof

Suppose *v* is a solution of the problem (1.1). First, we prove *v* is nonnegative. Define the test function $\varphi_{-}=\min\{0,v\}$ and substitute in the integral formula of Definition 2.1. We can obtain

$$\frac{1}{2} \bigl\Vert \varphi_{-}(t) \bigr\Vert ^{2}_{2,\Omega} \leq- \int _{Q_{t}} \bigl(|\nabla\varphi_{-}|^{p}+ \lambda|x|^{\alpha}\chi_{\{\varphi _{-}>0\}}{\varphi_{-}}^{1-\beta} \bigr)\,dz \leq0 $$

in $Q_{t}=(0,t)\times\Omega$ for every $t< T^{\ast}$, through the definition of $g_{\varepsilon,\eta}$ and $\varphi_{-}$. Then $v\geq 0$ a.e. in $Q_{t}$ for every $t< T^{\ast}$.

Next, we prove $v\leq M$. By Theorem 3.1, problem (1.1) has a local in time solution *v*, then $\partial_{t}v-\Delta_{p}v\leq0$ in $L^{p'}(0,T;W^{-1,p'}(\Omega))$. Define the function $\Psi(t)=Ke^{t}$, where $K=\|u_{0}\|_{\infty,\Omega}$. It’s easy to see that

3.5 $$ \textstyle\begin{cases} \partial_{t}\Psi-\Delta_{p}\Psi=Ke^{t}\geq0 \quad \mbox{in } (0,T]\times\Omega, \\ \Psi\geq\|u_{0}\|_{\infty,\Omega}\quad \mbox{in } \Omega,\qquad \Psi>0 \quad \mbox{on } \Gamma. \end{cases} $$

For every $\varphi\in L^{p}(0,T;W_{0}^{1,p}(\Omega))$, we have

$$\int_{Q_{T}} \bigl\{ \partial_{t}(v-\Psi)\varphi+\bigl(| \nabla v|^{p-2}\nabla v-|\nabla\Psi|^{p-2}\nabla\Psi\bigr)\cdot \nabla\varphi \bigr\} \,dz\leq0. $$

Letting $\varphi_{+}:=\max\{0, v-\Psi\}\in L^{p}(0,T;W_{0}^{1,p}(\Omega ))$ and using the inequality

$$\bigl(|\xi|^{p-2}\xi-|\eta|^{p-2}\eta \bigr)\cdot(\xi-\eta ) \geq0, $$

we derive

$$\frac{1}{2} \bigl\Vert \varphi_{+}(t) \bigr\Vert ^{2}_{2,\Omega} \leq0, $$

so $\varphi_{+}=0$ a.e. in $Q_{T}$. Choosing $L=1+\|u_{0}\|_{\infty,\Omega }$, and fixing *T* by the relation

$$L\geq\Psi(T) \quad \Leftrightarrow\quad T=\ln \biggl(1+\frac {1}{\|u_{0}\|_{\infty,\Omega}} \biggr), $$

we have $0\leq v(x,t)\leq L$ a.e. in Ω for every $t\in[0,T]$. Then, taking $v(x,t)$ for the initial datum and repeating the comparison procedure with the new function

$$\Psi(t)= \bigl\Vert v(T) \bigr\Vert _{\infty,\Omega}e^{(t-T)},\qquad L'=1+ \bigl\Vert v(T) \bigr\Vert _{\infty,\Omega}, $$

we extend $v(x,t)$ to $\Omega\times[T,T']$, where $T'$ and $L'$ can be obtained by the above arguments, and conclude that $0\leq v(x,t)\leq L'$ for a.e. $x\in\Omega$ and $t\in[T,T']$. We continue this process until $(0,T^{\ast})$ is exhausted. This completes the proof of Theorem 3.2. □

### Theorem 3.3

*Let the conditions of Theorem *3.2 *be satisfied*. *Then the solution* *v* *of* (1.1) *is global in time*. *Moreover*, *for every* $T>0$, *v* *satisfies* $0\leq v\leq M$ *a*.*e*. *in* $Q_{T}$, *where* $M=M (p,\|u_{0}\|_{\infty,\Omega},\lambda_{1} )>0$.

By Theorem 3.2, we easily conclude that Theorem 3.3 can be established. Also, by the regularization arguments as when proving Theorem 3.4 in [22], we can derive the following theorem of higher regularity of solutions to problem (1.1). Here we state these results and omit the details (cf. [22]).

### Theorem 3.4

*Let the conditions of Theorem *3.2 *be fulfilled*. *If we add the hypothesis* $u_{0}\in W_{0}^{1,p}(\Omega)$, *then* $u\in \mathscr {U}$. *Moreover*, *for a*.*e*. $t\in(0,T^{\ast})$, *we have*

3.6 $$\begin{aligned}& \Vert \partial_{t}u \Vert ^{2}_{2,Q_{t}}+ \frac{1}{p} \bigl\Vert \nabla u(t) \bigr\Vert ^{p}_{p,\Omega} +\lambda \int_{\Omega}\int_{0}^{u(t)}|x|^{\alpha}\chi_{\{s>0\}}s^{-\beta } \,ds\,dx \\& \quad \leq \frac{1}{p} \Vert \nabla u_{0} \Vert ^{p}_{p,\Omega} +\lambda \int_{\Omega}\int_{0}^{u_{0}}|x|^{\alpha}\chi_{\{s>0\}}s^{-\beta} \,ds\,dx. \end{aligned}$$

## Complete quenching in finite time

In this section, following the idea of [16, 22] (see also the book [1]), we discuss the complete quenching phenomenon by using the energy methods and give the proof of Theorem 2.2. We here note that Díaz [16] has extended the energy method to the study of the free boundary generated by the solutions of more general semilinear or quasilinear parabolic problems of quenching type, which involve a negative power of the unknown in an equation like (1.1).

Define the energy function $J(t)= \|u(t) \|^{2}_{2,\Omega}$. In the following, we first derive the energy equality and ordinary differential inequality satisfied by $J(t)$.

From (2.1), we have the following equality for $t_{1}$, $t_{2}\in[0,T]$:

4.1 $$ \frac{1}{2} \bigl\Vert u(t_{2}) \bigr\Vert ^{2}_{2,\Omega}-\frac{1}{2} \bigl\Vert u(t_{1}) \bigr\Vert ^{2}_{2,\Omega}+ \int_{t_{1}}^{t_{2}} \int_{\Omega}\bigl( \vert \nabla u \vert ^{p}+ \lambda|x|^{\alpha}u^{1-\beta} \bigr)\,dz=0. $$

Letting $t_{1}=t$, $t_{2}=t+h$ with *t*, $t+h\in[0,T]$, we can rewrite (4.1) as

$$\frac{1}{2h} \bigl\Vert u(t+h) \bigr\Vert ^{2}_{2,\Omega}- \frac{1}{2h} \bigl\Vert u(t) \bigr\Vert ^{2}_{2,\Omega}+ \frac{1}{h} \int_{t}^{t+h} \int_{\Omega}\bigl( \vert \nabla u \vert ^{p}+ \lambda|x|^{\alpha}u^{1-\beta} \bigr)\,dz=0. $$

Since $u\in \mathscr {U}$ and it satisfies (2.1), we know that

$$\int_{\Omega}\bigl(|\nabla u|^{p}+\lambda|x|^{\alpha}u^{1-\beta} \bigr)\,dx\in L^{1}(0,T). $$

Applying the Lebesgue differentiation theorem for a.e. $t\in(0,T)$, we have

$$ \lim_{h\rightarrow0}\frac{1}{h} \int_{t}^{t+h} \int_{\Omega}\bigl( \vert \nabla u \vert ^{p}+ \lambda \vert x \vert ^{\alpha}u^{1-\beta} \bigr)\,dz= \int_{\Omega}\bigl( \bigl\vert \nabla u(t) \bigr\vert ^{p}+\lambda \vert x \vert ^{\alpha}u^{1-\beta}(t) \bigr)\,dx. $$

Using (4.1), we get the following energy equality for a.e. $t\in(0,T)$:

4.2 $$ \frac{1}{2}\frac{d}{dt} \bigl( \bigl\Vert u(t) \bigr\Vert ^{2}_{2,\Omega} \bigr)+ \int_{\Omega}\bigl( \bigl\vert \nabla u(t) \bigr\vert ^{p}+\lambda|x|^{\alpha}u^{1-\beta }(t) \bigr)\,dx=0. $$

By the definition of $J(t)$, we rewrite (4.2) in the following form for a.e. $t\in(0,T)$:

$$\frac{1}{2}J'(t)+ \int_{\Omega}\bigl( \bigl\vert \nabla u(t) \bigr\vert ^{p}+\lambda \vert x \vert ^{\alpha}u^{1-\beta}(t) \bigr)\,dx=0. $$

Setting $D=2\min\{1,\lambda\}$, we get the ordinary differential inequality

4.3 $$ J'(t)+D \int_{\Omega}\bigl( \bigl\vert \nabla u(t) \bigr\vert ^{p}+ \vert x \vert ^{\alpha}u^{1-\beta }(t) \bigr)\,dx \leq0. $$

To prove the differential inequality satisfied by $J(t)$ in Lemma 4.2, we will make use of the interpolation inequality with weights of Gagliardo–Nirenberg type (see [33]) as follows.

### Lemma 4.1

*Assume* *p*, *q*, *r*, *α*, *β*, *γ*, *a* *are real numbers*, *satisfying* $0< a<1$, $p, q\geq1$, $\frac{1}{p}+\frac {\alpha}{n}$, $\frac{1}{q}+\frac{\beta}{n}$, $\frac{1}{r}+\frac {\gamma}{n}>0$, $r\neq0$, *then*

$$\bigl\Vert |x|^{\gamma}D^{j}u \bigr\Vert _{L^{r}} \leq c \bigl\Vert |x|^{\alpha}D^{m}u \bigr\Vert ^{a}_{L^{p}} \bigl\Vert |x|^{\beta}u \bigr\Vert ^{1-a}_{L^{q}}, $$

*where* $j\geq0$, $m>0$ *are integers*, $j/m\leq a\leq1$, *and* $m-j-n/p$ *is not a nonnegative integer*.

### Lemma 4.2

*Assume that* $u\in \mathscr {U}$ *is a weak solution of problem* (1.1) *satisfying* (2.1). *Then the function* $J(t)$ *satisfies the differential inequality*

4.4 $$ \textstyle\begin{cases} J'(t)+KJ^{d}(t)\leq0, \quad \textit{a.e. } t\in(0,T),\\ J(0)= \|u_{0} \|^{2}_{2,\Omega}, \end{cases} $$

*with the constants* $K= (c^{-1}D^{\frac{a}{p}}(DM^{-\beta })^{1-a} )^{2d}$, $d=\frac{1}{2 (\frac{a}{p}+1-a )}\in(0,1)$, $M=\|u\|_{\infty,Q_{T}}$.

### Proof

Set $m=1$, $j=\alpha=\gamma=0$, $r=2$, $q=1$. Then applying Lemma 4.1 we can derive that for a.e. $t\in(0,T)$,

4.5 $$\begin{aligned}& D^{\frac{a}{p}} \bigl(DM^{-\beta} \bigr)^{1-a} \bigl\Vert u(t) \bigr\Vert _{2,\Omega} \\& \quad \leq D^{\frac{a}{p}} \bigl(DM^{-\beta} \bigr)^{1-a}c \bigl\Vert \nabla u(t) \bigr\Vert ^{a}_{L^{p}} \bigl\Vert |x|^{\alpha}u \bigr\Vert ^{1-a}_{L^{1}} \\& \quad = c \biggl(D \int_{\Omega} \vert \nabla u \vert ^{p}\,dx \biggr)^{\frac {a}{p}} \biggl(D \int_{\Omega}|x|^{\alpha}uM^{-\beta}\,dx \biggr)^{1-a} \\& \quad \leq c \biggl(D \int_{\Omega}|\nabla u|^{p}\,dx+D \int_{\Omega}|x|^{\alpha}uM^{-\beta}\,dx \biggr)^{\frac{a}{p}+1-a}. \end{aligned}$$

Since

$$\int_{\Omega}u(t)^{1-\beta}\,dx\geq M^{-\beta} \int_{\Omega}u(t)\,dx, $$

we obtain

$$\bigl(c^{-1}D^{\frac{a}{p}}\bigl(DM^{-\beta} \bigr)^{1-a} \bigr)^{2} J(t)\leq \biggl(D \int_{\Omega}\bigl\vert \nabla u(t) \bigr\vert ^{p} \,dx+ \int_{\Omega}|x|^{\alpha}u^{1-\beta}(t)\,dx \biggr)^{2 (\frac{a}{p}+1-a )}. $$

We complete the proof by plugging this inequality into (4.3). □

### Proof of Theorem 2.2

Now we will complete the proof of Theorem 2.2, which can be proved by the following lemma. □

### Lemma 4.3

*Assume* $J(t)$ *is a nonnegative function satisfying inequality* (4.4) *with* $d\in(0,1)$. *Then*

4.6 $$ J(t)=0,\quad \forall t\geq T_{\ast}, $$

*where* $T_{\ast}=J_{0}^{1-d}[K(1-d)]^{-1}$ *with* $J_{0}=J(0)$ *and* *K* *being defined in Lemma *4.2.

### Proof

Since (4.6) is surely true if $J_{0}=0$, so we just prove it for the case $J_{0}>0$. There exists an interval $(0,\tau)$ such that $J(t)>0$ for all $t\in[0,\tau)$ if $J_{0}>0$. For contradiction, we assume

$$\xi=\sup \bigl\{ \tau>0: J(t)>0, \forall t\in[0,\tau) \bigr\} >T_{\ast}. $$

Dividing both terms of inequality (4.4) by $J^{d}(t)$, we obtain

$$\frac{1}{1-d} \bigl(J^{1-d}(t) \bigr)'\leq-K. $$

Integrating it from 0 to *t* with $t\in(T_{\ast},\xi)$, we get

$$J^{1-d}(t)\leq J_{0}^{1-d}-K(1-d)t. $$

Since (4.4) is established, so $J'(t)\leq0$ for a.e. *t* and $J(t)$ is a nonincreasing function. On the other hand, $J(t)$ is nonnegative and $t\rightarrow J_{0}^{1-d}-K(1-d)t$ is monotone decreasing in *t*, thus

$$\forall t\geq T_{\ast}, \quad 0\leq J(t)\leq J_{0}^{1-d}-K(1-d)t< 0. $$

However, this is impossible unless $T_{\ast}\geq\xi$. Thus, $J(T_{\ast})=0$. □

## Numerical experiments

In this section, we give some numerical experiments which illustrate our theoretical results.

We consider the case of one space variable and mimic the numerical scheme in [41], and by the pdepe solver we convert equation (1.1) to ODEs using a second-order accurate spatial discretization based on a fixed interval of specified nodes. We refer the interested readers to [41], where the discretization method is described in detail.

We take $\Omega=[0,5]$ and $0=x_{1}< x_{2}<\cdots<x_{N}=5$ with $N=10$. By calling the pdepe function in Matlab, we can obtain the figures of numerical solution for $p=2$ and $p=4$, respectively. We know the solution will be quenching completely in finite time, through Theorem 2.2.

**Figure 1:**
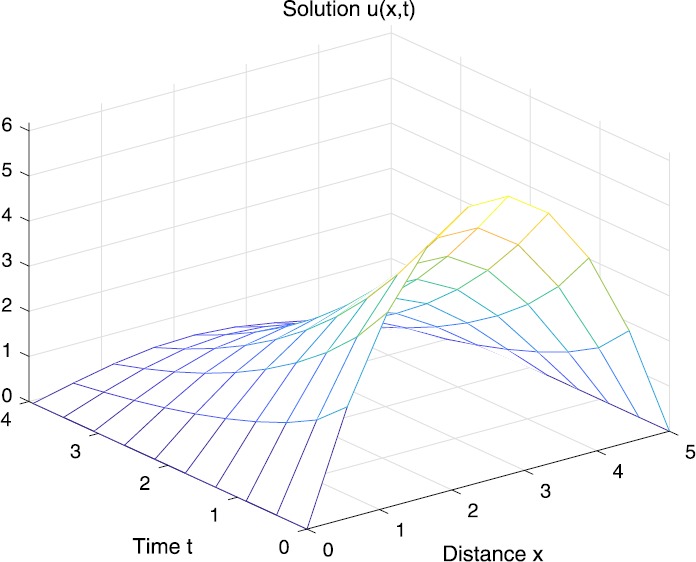
$p=2$, $\alpha=0.66$

**Figure 2:**
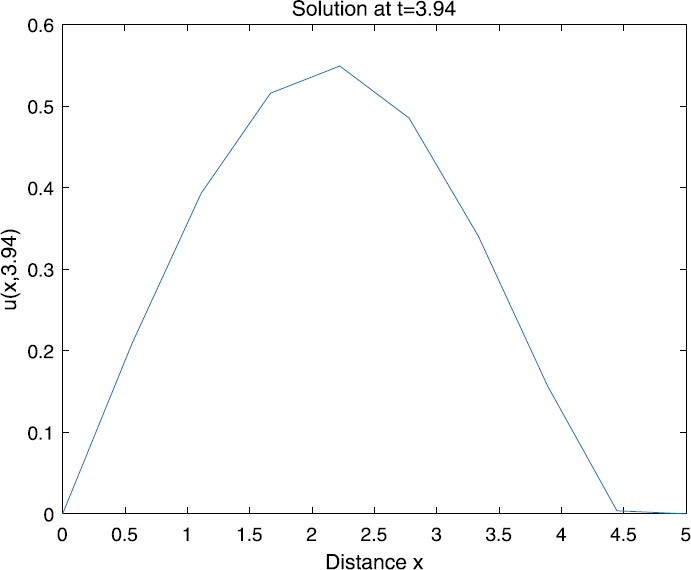
$p=2$, $\alpha=0.66$

**Figure 3:**
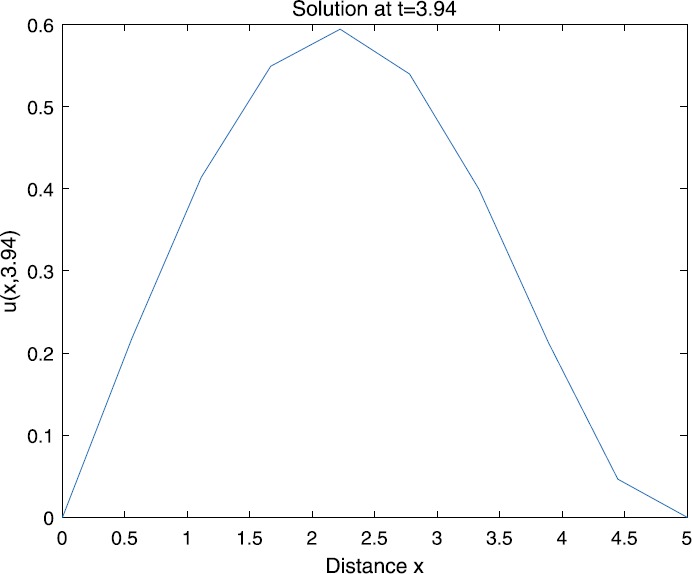
$p=2$, $\alpha=0.6$

**Figure 4:**
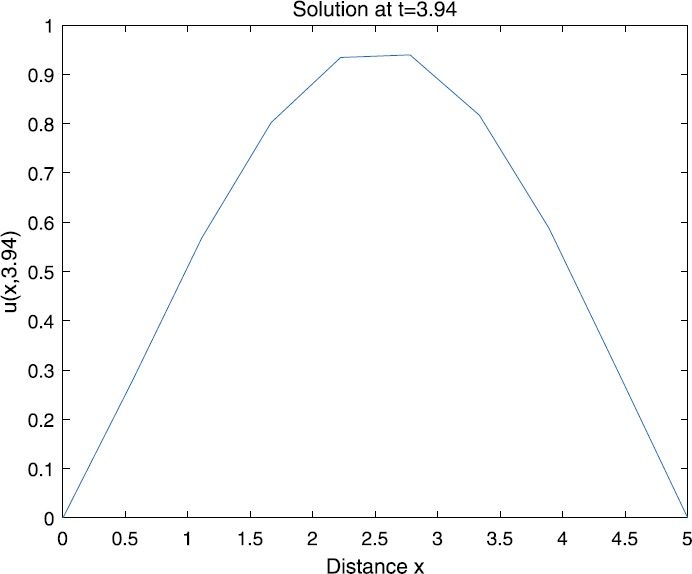
$p=2$, $\alpha=-0.1$

**Figure 5:**
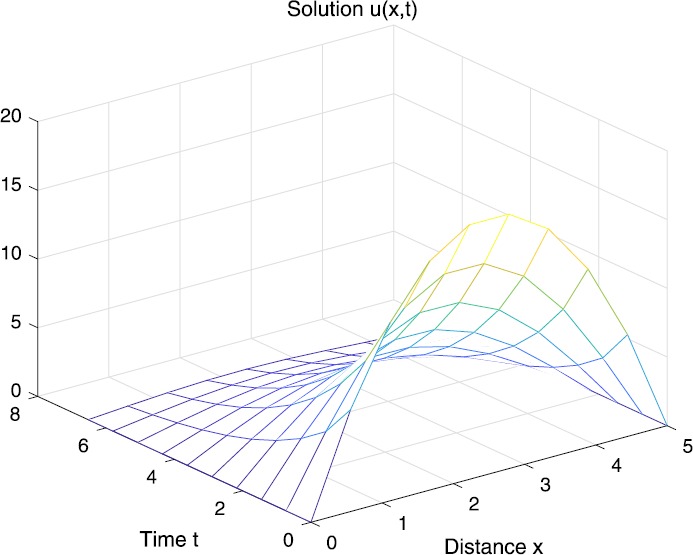
$p=4$, $\alpha=0.66$

**Figure 6:**
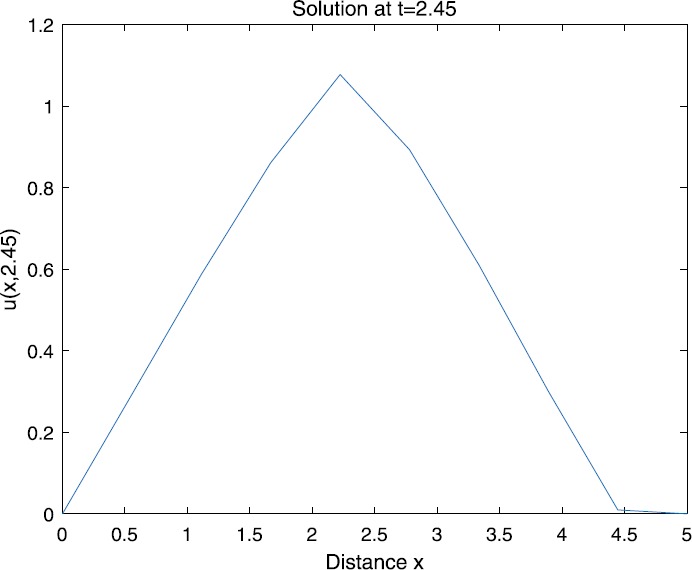
$p=4$, $\alpha=0.66$

**Figure 7:**
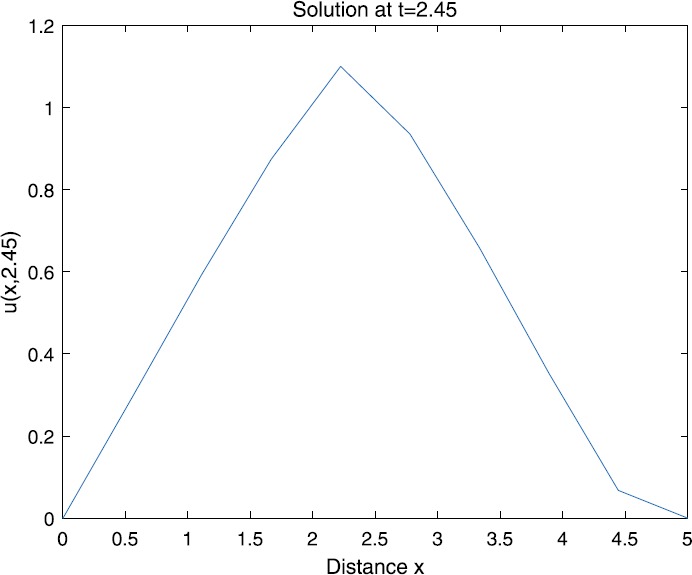
$p=4$, $\alpha=0.6$

**Figure 8:**
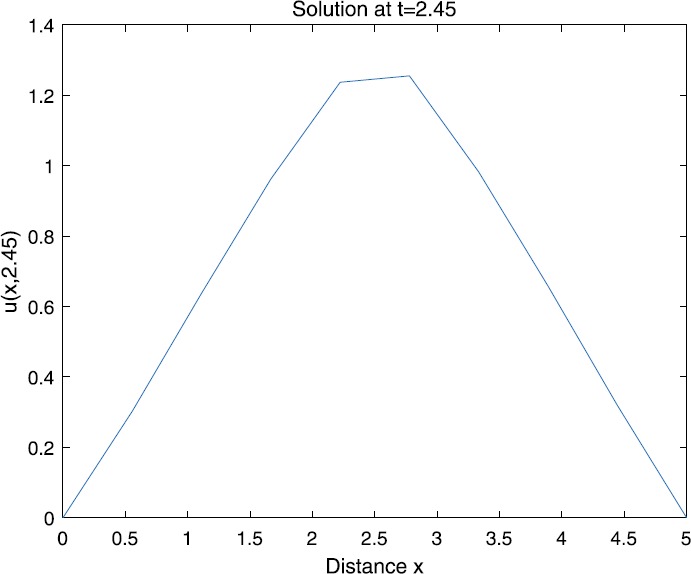
$p=4$, $\alpha=-0.1$

When $\beta=0.1$, $\lambda=0.2$ and $u_{0}=x(5-x)$, we can get the corresponding figures (see Figs. 1–8). When $p=2$ and $\alpha=0.66$, we can get the three-dimensional map, and obtain the corresponding sectional drawings for $\alpha=0.66, 0.6, -0.1$ when $t\sim3.94$ (Figs. 1–4). From Fig. 2, we know that the solution has been completely quenched in a small interval. According to Fig. 1, the solution will be quenching completely as time *t* passes. We can also get the figures when $p=4$ (Figs. 5–8). Choosing the same *β*, *λ*, *α*, $u_{0}$ and a different *p*, we know that the complete quenching time is also different. Figures 2–6 show that the complete quenching time decreases as *p* increases.

Theorem 2.2 and Lemma 4.3 show that the complete quenching time depends on $u_{0}$, *α*, *β*, *λ*, *p* and $|\Omega|$. Assuming *β* and *λ* remain fixed and choosing $u_{0}=3x(5-x)$, we can also get the complete quenching time (see Figs. 9–16).

**Figure 9:**
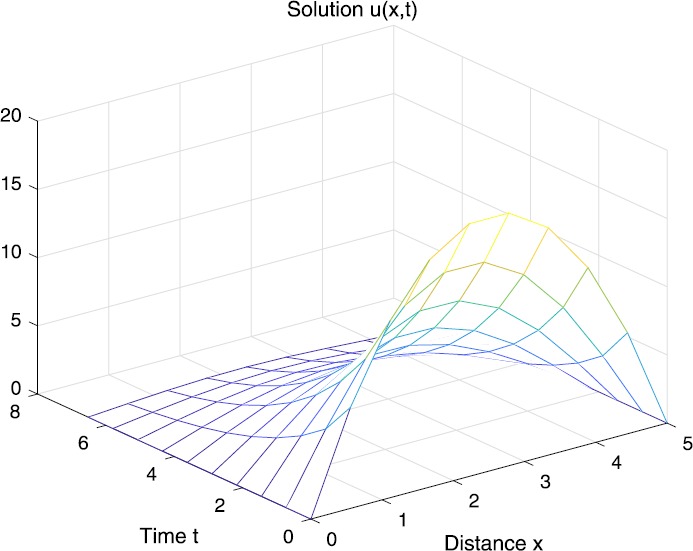
$p=2$, $\alpha=0.66$

**Figure 10:**
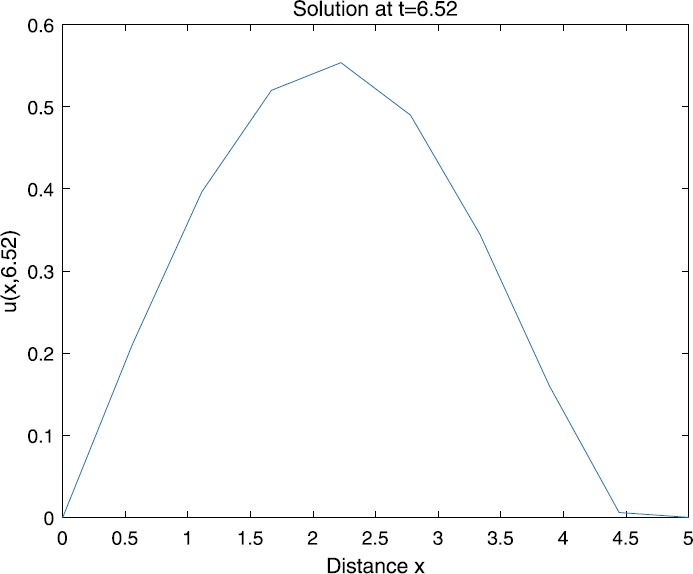
$p=2$, $\alpha=0.66$

**Figure 11:**
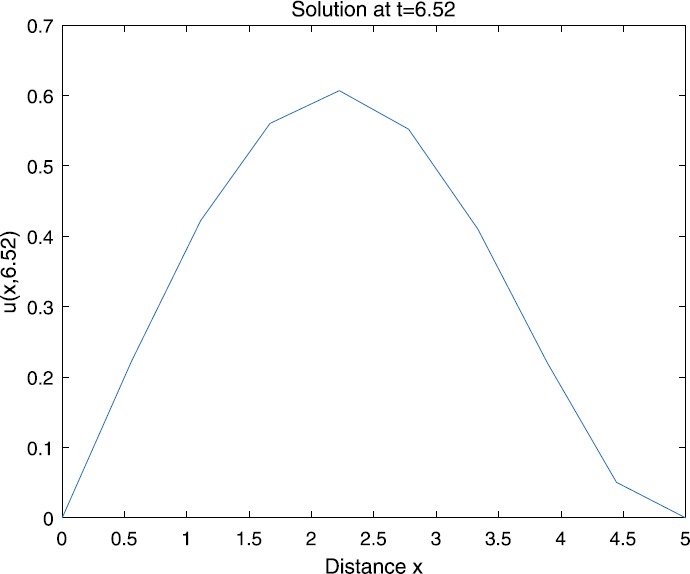
$p=2$, $\alpha=0.6$

**Figure 12:**
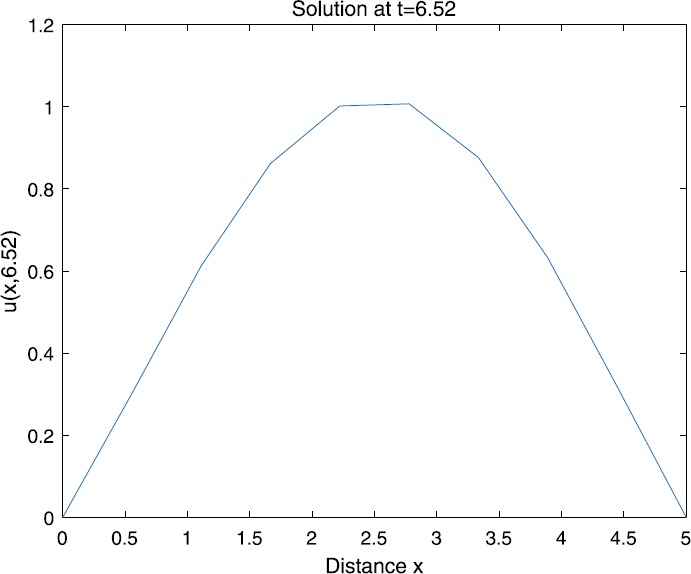
$p=2$, $\alpha=-0.1$

**Figure 13:**
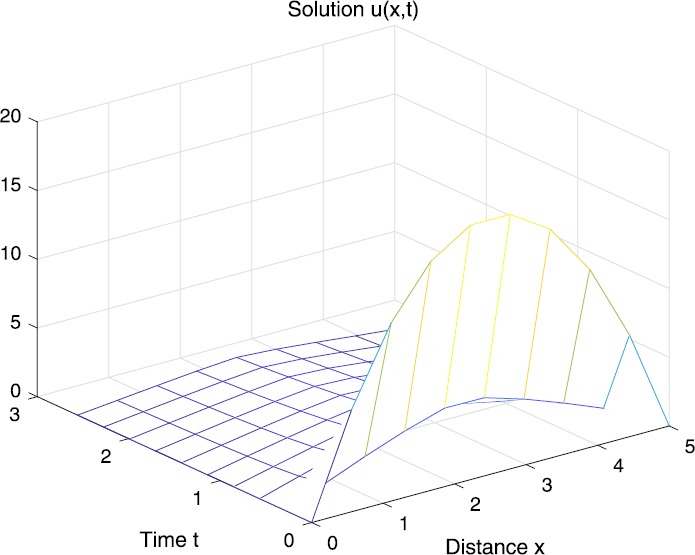
$p=4$, $\alpha=0.66$

**Figure 14:**
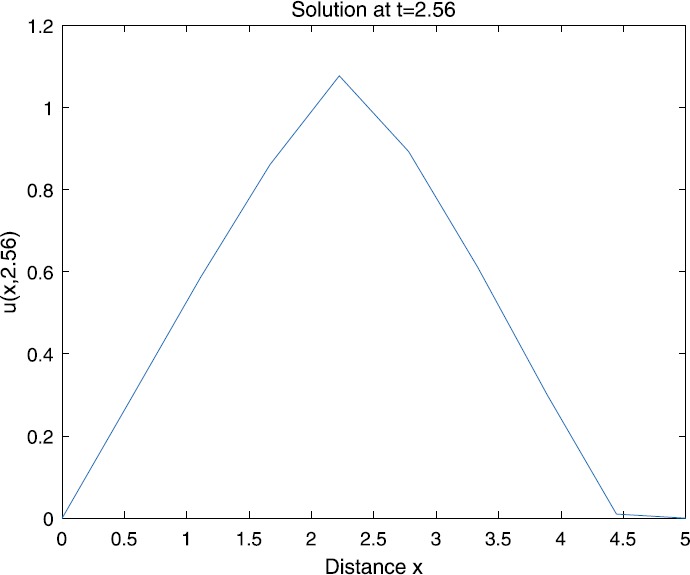
$p=4$, $\alpha=0.66$

**Figure 15:**
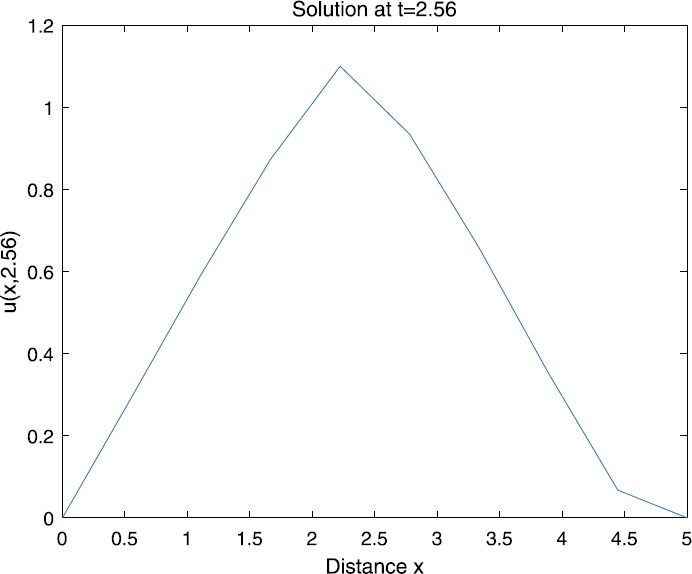
$p=4$, $\alpha=0.6$

**Figure 16:**
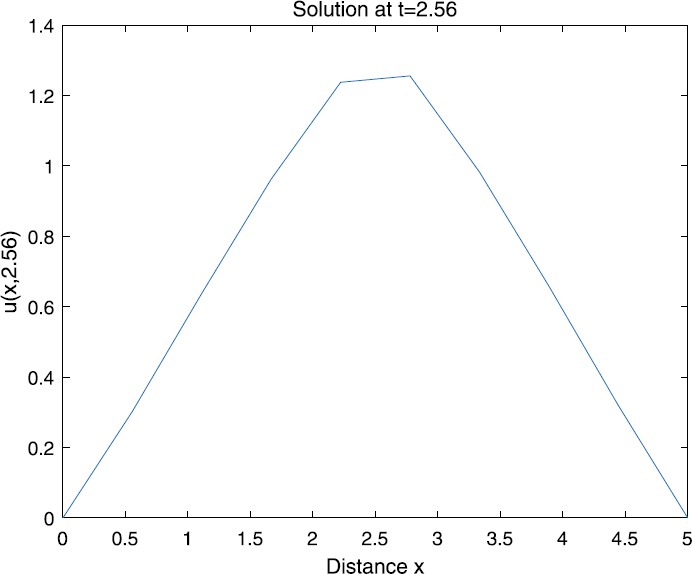
$p=4$, $\alpha=-0.1$

According to Figs. 1–16, we find that, as $u_{0}$ gets larger, the complete quenching time will be also longer. Moreover, from the figures of $\alpha=-0.1, 0.6, 0.66$, we know that the complete quenching phenomenon will occur when *α* increases to some critical value, for example, $\alpha\sim0.66$ in above numerical experiments.

### Remark 5.1

In this section, we only show the complete quenching phenomenon of numerical solutions by choosing some special parameters of *λ*, *β*, *α*, *p* and certain initial data. In other words, the global weak solutions obtained in Theorem 2.2 are not unique, in general. When $p=2$, $\lambda=1$ and $\alpha=0$ are taken in equation (1.1), Winkler [43] has shown that, for any *n* and *β*, the nonuniqueness holds at least for some nonnegative boundary and initial data. We suspect that similar results would still hold for the quasilinear equation (1.1). We leave it to the interested readers as an open question.
